# TNFα-Damaged-HUVECs Microparticles Modify Endothelial Progenitor Cell Functional Activity

**DOI:** 10.3389/fphys.2015.00395

**Published:** 2015-12-22

**Authors:** Carlos Luna, Andrés Carmona, Matilde Alique, Julia Carracedo, Rafael Ramirez

**Affiliations:** ^1^Instituto Maimónides de Investigación Biomédica de Córdoba, Hospital Universitario Reina Sofía/Universidad de Córdoba, Cellular Damage in Chronic InflammationCórdoba, Spain; ^2^RETICs Red Renal (Instituto de Salud Carlos III)Madrid, Spain; ^3^Departamento Biología de Sistemas, Facultad de Medicina y Ciencias de la Salud, Universidad de AlcaláAlcalá de Henares, Spain

**Keywords:** endothelial microparticles, endothelial progenitor cells, angiogenesis, TNFalpha, HUVECs

## Abstract

Endothelial progenitor cells (EPCs) have an important role in the maintenance of vascular integrity and homeostasis. While there are many studies that explain EPCs mechanisms action, there are few studies that demonstrate how they interact with other emerging physiological elements such as Endothelial Microparticles (EMPs). EMPs are membranous structures with a size between 100 and 1000 nm that act as molecular information transporter in biological systems and are known as an important elements in develop different pathologies; moreover a lot of works explains that are novel biomarkers. To elucidate these interactions, we proposed an *in vitro* model of endothelial damage mediated by TNFalpha, in which damaged EMPs and EPCs are in contact to assess EPCs functional effects. We have observed that damaged EMPs can modulate several EPCs classic factors as colony forming units (CFUs), contribution to repair a physically damaged endothelium (wound healing), binding to mature endothelium, and co-adjuvants to the formation of new vessels *in vitro* (angiogenesis). All of these in a dose-dependent manner. Damaged EMPs at a concentration of 10^3^ MPs/ml have an activating effect of these capabilities, while at concentrations of 10^5^ MPs/ml these effects are attenuated or reduced. This *in vitro* model helps explain that in diseases where there is an imbalance between these two elements (EPCs and damaged EMPs), the key cellular elements in the regeneration and maintenance of vascular homeostasis (EPCs) are not fully functional, and could explain, at least in part, endothelial dysfunction associated in various pathologies.

## Introduction

Several studies have shown that most damaged cells produce and release microparticles (MPs) and act as signaling factors that elicit a coordinated cellular response to the injury (Soriano et al., 2014). These MPs are membranous bodies ranging from 0.2 to 1 μm in size and are present in different biological fluids. The quantification of these particles in plasma could be used as a diagnostic tool because pathological conditions often result in an increased number of MPs due to poor endothelial cell function (Diehl et al., 2008).

There is a physiological mechanism responsible for the repair of the endothelium to prevent the loss of its integrity produced by vascular disease (Shantsila et al., 2007). This repair process involves the recruitment of endothelial progenitor cells (EPCs) from the bone marrow. A correlation has previously been found between the numbers of MPs and EPCs in the peripheral blood (Burger and Touyz, 2012). Initially, the proliferation and migration of adjacent endothelial cells had been identified as the endothelial repair factor; subsequent studies described a mechanism for endothelial structure maintenance that was associated with the capacity of circulating EPCs to differentiate and repair the damaged endothelial tissue (Asahara et al., 1997). This mechanism is supported by the fact that dysfunction of the endothelium is associated with compromised EPC function (Peng et al., 2015).

Due to the importance of this endothelial damage-repair mechanism in the maintenance of vascular homeostasis, it is logical to think the existence of a close communication between the damaged endothelial cells and EPCs. What these signals may be and when they are activated have not yet been elucidated and could involve several factors (Deregibus et al., 2007).

Previous studies performed by our group indicate that plasma MPs, in both healthy controls and chronic kidney disease, stimulate the activity of EPCs (Soriano et al., 2014). However, we do not know whether this effect is due to the activity of all circulating MPs or is specific to endothelial MPs (EMPs). Therefore, it is important to transfer these results to an *in vitro* endothelial model in which the only factor utilized is derived from the endothelium.

Despite the previously described findings, we have not been able to find other studies that analyzed whether damaged endothelial cells and subsequent EMP production are capable of modulating the repair activity promoted by EPCs; we believe that this question must be addressed to improve our knowledge of the physiological mechanisms involved in maintaining endothelial integrity. Therefore, we performed this study to determine the EMP production capacity of a damaged endothelial cell model and whether these EMPs are able to stimulate the functional activity of EPCs in a dose-dependent manner.

## Materials and methods

### Human umbilical vein endothelial cell culture

Human umbilical vein endothelial cells (HUVECs) were obtained from Cell Systems (Clonetics, Solingen, Germany) and cultured at 37°C in a 5% CO_2_ atmosphere in standard endothelial cell basal medium (EBM, CAMBREX Bios Science, Walkersville, Inc., Walkersville, MD, USA) plus endothelial cell-growth medium supplements (EGM, CAMBREX) and 10% fetal calf serum (FCS, Invitrogen; Molecular probes, Eugene, OR, USA). First-passage cryopreserved HUVECs were grown and serially passaged; the number of population doublings (PDs) was calculated using the formula PD = (ln [number of cells harvested]–ln [number of cells seeded])/ln2.

To stimulate HUVECs, the cells were cultured for 24 h at each passage with tumor necrosis factor alpha (TNFalpha, SIGMA-ALDRICH), 10 ng/ml. The optimal concentration and time of culture with TNFalpha were based on preliminary studies from concentration and time-dependence studies (Majewska et al., 1997).

### Endothelial activation by TNFalpha

Cells were treated with 10 ng/ml TNFalpha (Majewska et al., 1997) for various incubation times (24, 48, and 72 h). The cells were harvested by mechanical disruption and separated by centrifugation. After washing twice with sterile phosphate buffered saline (PBS), the cells were labeled with Monoclonal antibodies (mAbs) against PECAM [phycoerythrin (PE)-labeled mAb, CD106, BD Pharmingen™, ref555647] and ICAM [fluorescein 5′-isothiocyanate (FITC)-labeled mAb, CD54, Invitrogen, ref MHCD5401] for 20 min in the dark. Apoptosis was evaluated by the percentage of annexin V+/PI+ (Propidium Iodide) cells. Endothelial cell proliferation was quantified by the expression of proliferating cell nuclear antigen (PCNA) (FITC-labeled mouse anti-human PCNA, BD Pharmingen™, ref 556030) according to the manufacturer's instructions. Analysis of adhesion markers, proliferation and apoptosis were performed via flow cytometry (Accuri C6, BD Biosciences, USA).

### MPs isolation and quantification

The supernatant of TNFalpha-treated (10 ng/ml) HUVECs was collected at 24 h and centrifuged at low speed (2500 rpm, 10 min, 4°C) to eliminate cell debris. Then, the supernatant was centrifuged several times at 13000 rpm at 4°C for 10 min to concentrate the MPs. The supernatant were not discarded, to produce EMP-Free supernatant. Flow cytometry (Accuri C6 flow cytometer, Becton Dickinson, USA) was used to determine the quantity of MPs secreted by the endothelial cells in the presence of TNFalpha at 24 h. As an internal reference for size and complexity, 1-μm diameters Flow-Count Fluoro-spheres (Beckmann Coulter) were used. In addition, the MP population was restricted by size [forward-scatter area (FSC-A) 10^5.5^–10^6^] and complexity [side-scatter area (SSC-A) 10^4^–10^6^]. Afterward, direct relationships were used to determine the volumes required to treat the EPC culture (10^3^ and 10^5^ MPs/ml).

### MP distribution on endothelial cells

EMPs suspended in 60 μl of PBS were labeled with 2 μl of Cell Tracker™ CM-DiI (Molecular Probes, Life Technologies, USA) for 2 h at 37°C in darkness. The EMPs were then washed two times in sterile PBS (2 × 10,000 g, 4°C) to remove excess probe. Finally, the labeled and washed EMPs were again suspended in 60 μL of sterile PBS and distributed to HUVEC cultures.

HUVECs (10^4^ cells/glass coverslip) were treated with labeled MPs for 24 h to visualize the microparticles on endothelial cells. The cells were then fixed with 2% paraformaldehyde for 10 min, permeabilized with 0.1% (v/v) Triton X-100 in PBS for 10 min, washed with PBS, blocked with 4% bovine serum albumin (BSA) for 1 h at room temperature and incubated overnight at 4°C with anti-phalloidin Ab (1:200 dilution). Next, the cells were incubated at 37°C with α-mouse-Alexa-Fluor® 633 (1:2000) and α-rabbit-Alexa-Fluor® 488 (1:2000) for 1 h in the dark. The coverslips were washed and mounted with ProLong Gold antifade reagent with 4′-6-diamidino-2-phenylindole (DAPI) (Invitrogen Eugene, Oregon). Detection was performed by confocal laser scanning microscopy (LEICA TCS-SL, Heidelberg, Germany).

### EPC culture

Peripheral blood mononuclear cells from healthy donors were isolated by density gradient centrifugation (Lymphoprep, Axis-Shield PoC, Oslo, Norway), washed with PBS (GIBCO, Invitrogen, Carlsbad, CA) plus 20% FBS, and suspended in endothelial cell basal medium-2 (EBM-2) (Lonza, Allendale, NJ, USA) supplemented with single aliquots of endothelial cell growth medium (EGM-2) (VEGF, human EGF-B, recombinant IGF-1, human EGF, heparin, ascorbic acid, and GA-100, Lonza) and 15% autologous plasma. Mononuclear cells were plated onto fibronectin (Biocoat, BD Biosciences, Franklin Lakes, NJ, USA) in coated, six-well plates at a density of 5 × 10^6^ cells/well. We then incubated the fibronectin-coated plates at 37°C in a 5% CO_2_ atmosphere; 4 days later, we removed the cells in suspension, and the fraction of attached cells was cultivated with EBM-2 supplemented with 15% autologous plasma. The medium was recharged every 2 days for 3–4 weeks. After this period, EPCs could be visualized with an optical microscope (OPTIKA Microscopes, Italy) in the form of colonies (colony forming units, CFUs). The EPC phenotype (CD34+CD133+VEGFR2+) was verified with a cellular purity of >90%.

### EPC proliferation activity—use of various doses of MPs obtained from endothelial cultures treated with TNFalpha for 24 h

EPCs enriched from cultures obtained with the method described in the previous section were used for treatment with different concentration of MPs from TNFalpha-treated HUVECs. On 7th day EPCs cultures, the two different concentrations of MPs (10^3^ and 10^5^ MPs/ml) were added. The medium and the two different stimuli were renewed every 2 days until the EPCs culture end (28th day). On day 28, the number of adherent cell colonies was counted by 3 observers using an inverted fluorescence microscope (OPTIKA Microscopes, Italy) in 10 random high-power fields.

### Wound assay

To evaluate the wound healing capacity of the treated EPCs in a mature endothelial cell monolayer, we inflicted a wound by physical damage perpendicular to the well axis when the cells were at 70–80% confluence. Afterward, 15000 treated EPCs were added to the wounded endothelial cell monolayer, and the healing process was monitored over 2-h periods until reaching 24 h. At 24 h, we observed the extent of wound healing with regard to the EPCs treated with 10^3^ MPs/ml. At the indicated intervals, photographs were taken with an optical inverted microscope (OPTIKA Microscopes, Italy), and the images were analyzed using the included software (Optika Vision Pro, Italy); the percentage of the closed area compared with the initial wound area was quantified.

### EPC adhesion over a HUVEC monolayer

To observe the magnitude to which MP-treated EPCs can adhere to an endothelial monolayer, EPCs were harvested by mechanical disruption with a mini-scrapper, and labeled with Cell Tracker™ CM-DiI (Molecular Probes, Life Technologies, USA) at 37°C for 2 h in the dark and in appropriated medium (EBM-2, Lonza, USA) which enabled the detection of the EPCs on an endothelial cell monolayer via fluorescence. For this purpose, EPC cells that were treated with different doses of TNFalpha MPs and previously labeled with Cell Tracker™ CM-DiI were added (50,000/well) to 6-position culture plates containing endothelial cells at 70–80% confluence, and the co-cultures were stabilized for 24 h. The number of adherent fluorescent EPCs in 10 random high-power fields was quantified using IMAGEj (Eclipse Ti-S, Nikon Instruments Europe B.V., Badhoevedorp, The Netherlands). The quantification is related to the randomized number of fluorescent pixels.

### Angiogenesis assay in matrigel

To evaluate the effect of treated EPCs on endothelial cells, angiogenesis experiments were designed in 15-well plates (IBIDI) coated with 10 μl of Matrigel (Corning). For the angiogenesis assay, a total of 2500 cells/well were plated, of which 50% were EPCs obtained from MP-treated cultures, and the remaining 50% were mature endothelial cells (HUVECs) at low passage stages (3–4), with their respective treatments, as well as an internal positive control, VEGF (50 ng/ml). The formation of vascular structures was monitored over a period of 2–3 h. Once this period had elapsed, photographs were taken with an optical inverted microscope (Optika microscopia, Italy), and an automated analysis was performed with the IMAGEj software. Two parameters were taken for the quantification experiments (total master segments length and NB master junctions).

### Statistical analysis

Each sample was analyzed in triplicate, and each experiment was performed at least three times. The data are presented as the means ± SD, and analysis of variance (ANOVA) with a Bonferroni *post-hoc* correction was applied for the statistical analysis. The visual counts via microscopy were performed by three independent researchers. The statistical analyses and the creation of images were performed using the program Graph Pad Prism 5.0.

## Results

### TNFalpha induces endothelial damage after 48 h of treatment

**Figure 1:**
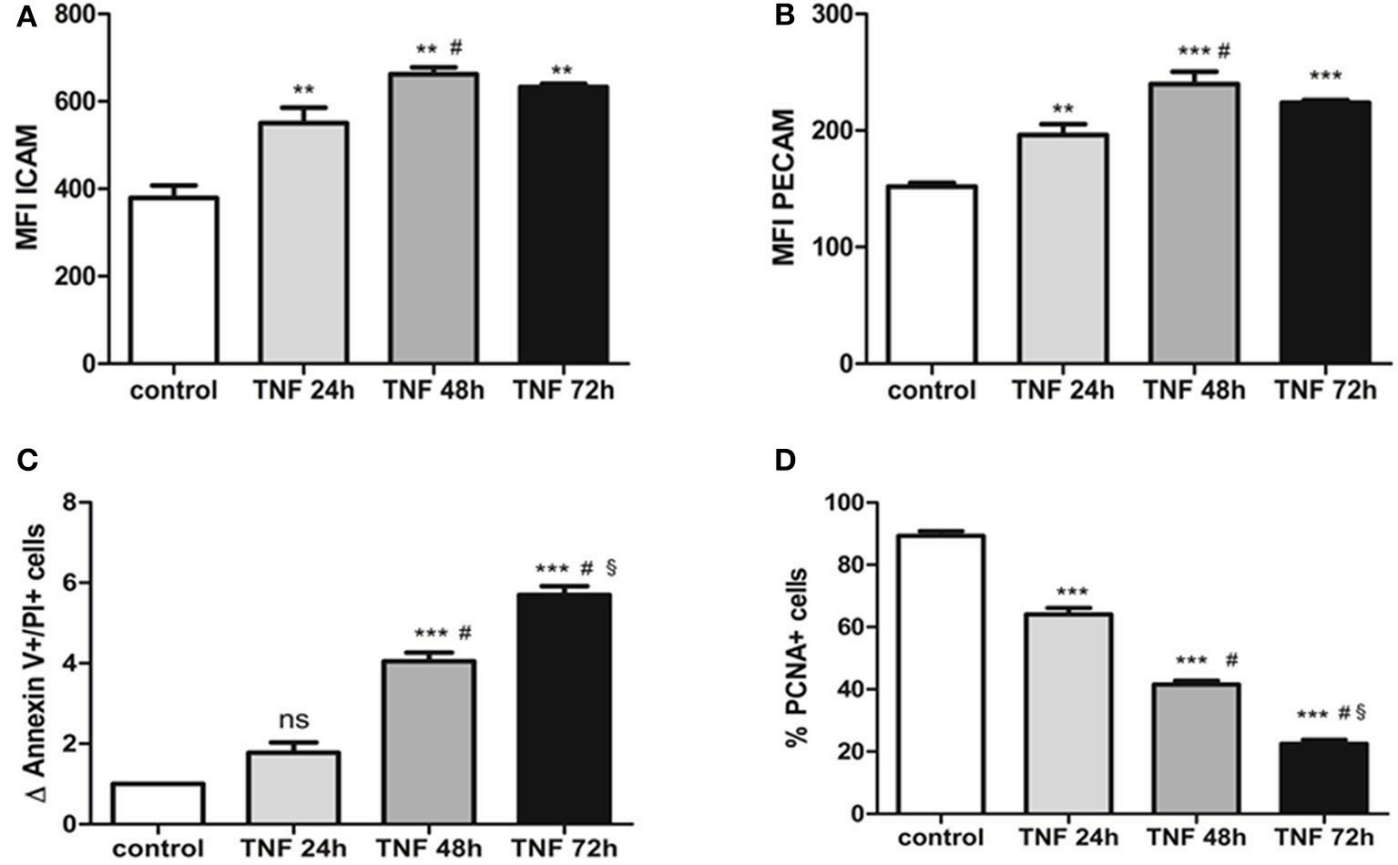
**(A)** ICAM expression obtained by flow cytometry of HUVECs treated with 10 ng/ml TNFalpha at different time points. The expression is quantified by changes in the mean fluorescent channel (MFC). **(B)** PECAM expression obtained by flow cytometry on HUVECS treated with 10 ng/ml TNFalpha at different time points. Expression units are similar to ICAM (MFC). **(C)** Differences in total numbers of annexin V+/IP+ cells (late apoptosis) observed via flow cytometry in cultures treated with TNFalpha at different time points. These values are compared with the control culture, in which the number of apoptotic cells is set to 1. **(D)** Decrease in the number of PCNA+ cells (proliferating cells) in cultures treated with TNFalpha at different time points. ^*^vs. control, ^#^vs. TNFalpha 24 h, ^§^ vs. TNFalpha 48 h. ^**^p < 0.05; ^***^p < 0.001.

We determined the extent of the damage produced by the pro-inflammatory cytokine TNFalpha. For this purpose, we started with a concentration known to modulate the expression of adhesion molecules (Majewska et al., 1997) and evaluated different treatment times to determine when the endothelial cells are activated without leading to cell damage. We observed that after 24 h of TNFalpha treatment, there is a significant increase in the mean fluorescence channel (MFC) for the ICAM1 molecule compared to 0 h (550.30 ± 35.61 vs. 378.90 ± 28.82; *p* < 0.05) (Figure 1). In a similar manner, the MFC for the PECAM molecule increases significantly at 24 h (196.2 ± 9.2 vs. 151.9 ± 3.17; *p* < 0.05) (Figure 1). At 48 h of treatment with TNFalpha, there is a more acute increase in the MFC for these two membrane molecules (Figure 1). However, at 72 h of treatment, the MFC for both markers decreases compared with 24 and 48 h.

The quantity of apoptotic cells undergoes a slight increase after 24 h of culture (1.7 ± 0.2 vs. 1 “n-fold”; *p* = 0.4). This increment is high and statistically significant at 48 and 72 h. Last, cell proliferation decreases over time in response to TNFalpha, such that at 24 h there is a significant decrease in the number of proliferating cells. Moreover, as the culture advances, the quantity of proliferating cells diminishes to levels of 22% at 72 h (Figure 1).

Considering all of these time-response data, we chose use the MPs obtained from the TNFalpha treated endothelial cells at 24 h because there are signs of activation but not of cellular damage, which would be observed at other times (48 and 72 h).

### Endothelial cells release a higher number of MPs in the presence of TNFalpha at 24 h

**Figure 2:**
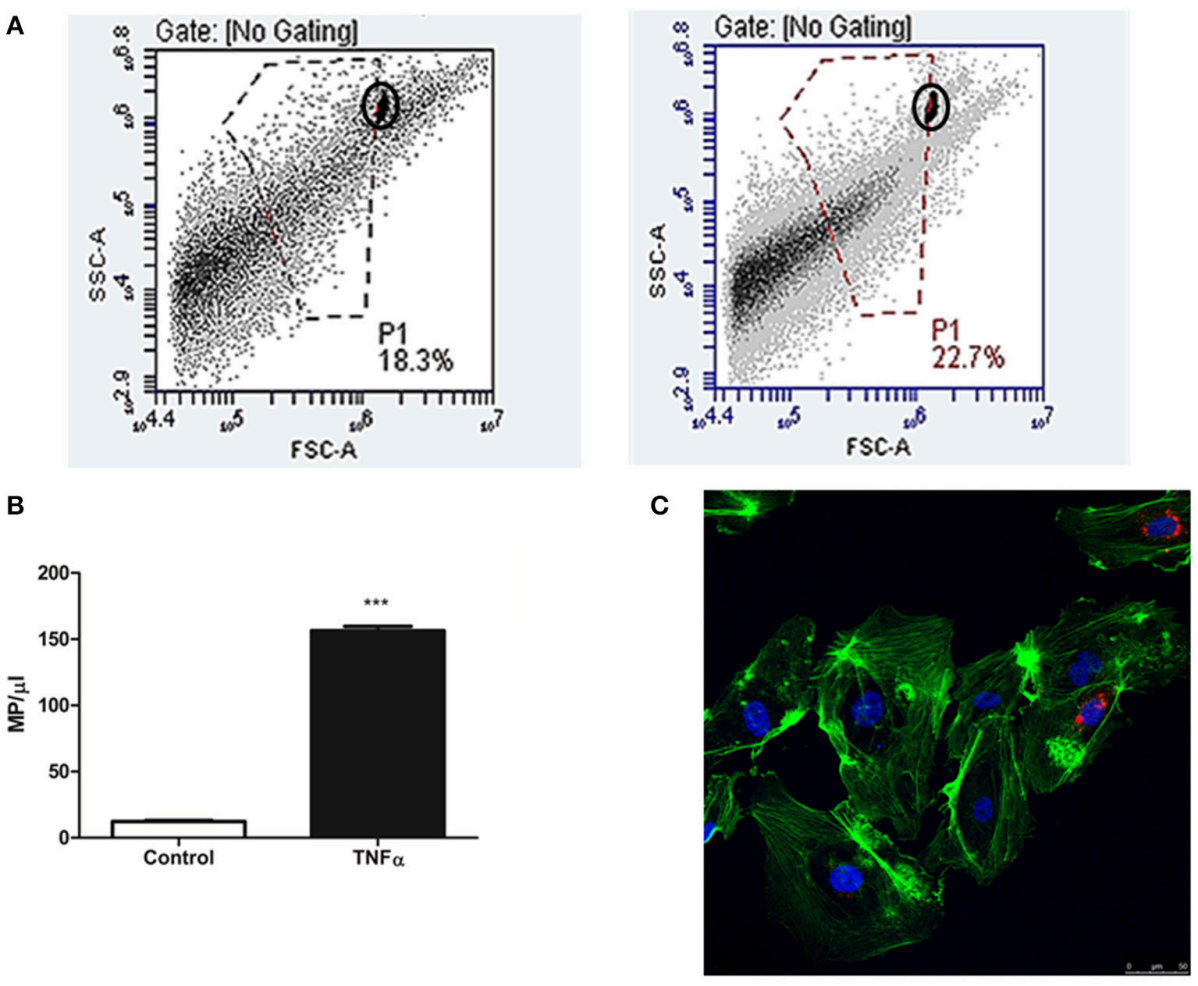
**(A)** Diagram of flow cytometry events that indicates the events corresponding to EMPs isolated from supernatants of HUVEC cultures treated with TNFalpha at 24 h (right) and control HUVECs (left). The MP population separated from background noise by manual gating, using 1-μm diameter fluorescent microspheres as a reference (circled). The X axis represents size (FSC-A), whereas the Y axis represents complexity (SSC-A), both in Log units. **(B)** Representative histogram of the quantity of isolated MPs secreted under these two conditions (control vs. TNFalpha 24 h). ^***^p < 0.001 vs. Control. **(C)** Confocal microscopy image at 24 h of HUVEC culture with 10^5^ MPs/ml. The MPs can be observed (marked in red with CellTracker™ CM-DiI) as accepted by the recipient cell, preferentially distributed within a perinuclear position.

We observed that the supernatants obtained after 24 h in culture have a significantly higher number of MPs/μl than those obtained from cells at the baseline state (156.44 ± 3.41 vs. 12.20 ± 0.86 MPs/μl; *p* < 0.001) (Figure 2). Moreover, we determined that these MPs are structures that transport information between HUVEC cells because we observed an incorporation of these MPs in 4–9 total cells/field on confocal microscopy images. Last, we also observed that the MPs are able to attain a perinuclear position (Figure 2).

### MPs from activated endothelial cells modulate the *in vitro* progression of EPC cultures

**Figure 3:**
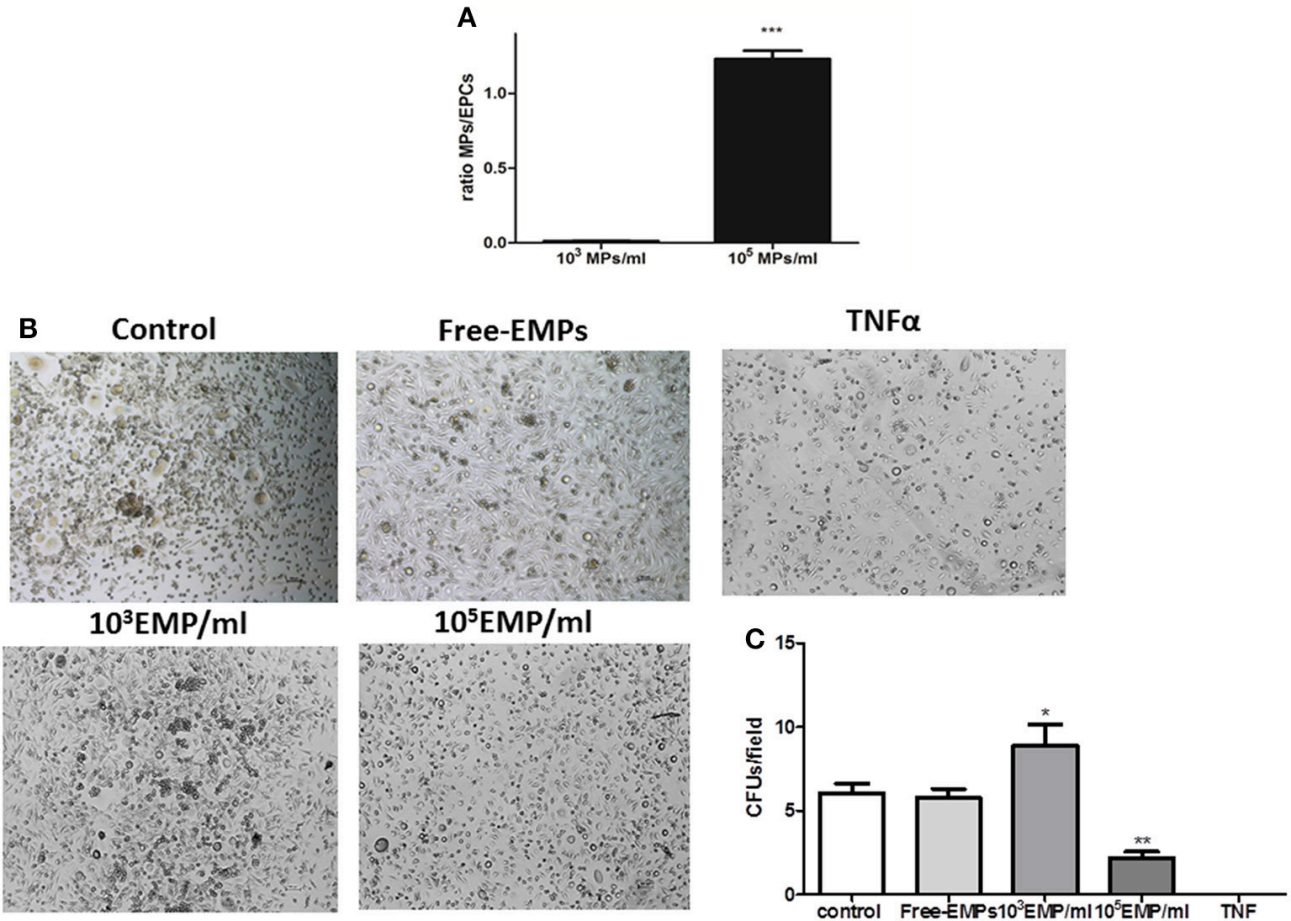
**(A)** Histogram of different ratios obtained after the MPS treatments in 7th day of EPCs cultures; ^***^vs. Control *p* < 0.001. **(B)** Optical inverted microscopy images (40 ×) of CFUs in EPC cultures at 25 days of culture with EMPs from TNFalpha-treated HUVECs at different concentrations. **(C)** Histogram of CFUs by field under different experimental conditions. It can be observed how TNFalpha treatment results in CFUs. ^*^vs. Control and FREE-EMPs, *p* < 0.05; ^**^vs. Control, FREE-EMPs, 10^3^EMP/ml, *p* < 0.001.

We observed that MPs obtained from endothelial cell cultures activated with TNFalpha for 24 h are capable of modulating the *in vitro* behavior of EPCs. After 7 days of culture, and after discarding cells that is not going to differentiate EPCs, a quantification of the number of cells per well was performed. The purification efficiency of cells obtained was 163,685 ± 12365 cells/well. These culture wells were treated with the two different doses of MPs (10^3^ MPs/ml and 10^5^ MPs/ml), in a total volume of 2 ml culture medium. The ratio obtained in the condition 10^3^ MPs/ml was 0.0123 ± 0.001 (< 2: 100 MPs/cell) and when using 10^5^ MPs/ml a ratio of 1.231 ± 0.02 was obtained (>1:1 MPs/cell) (Figure 3A).

After the end of the EPCs cultures (28th day) and when the EPCs are in contact with a concentration of 10^3^ MPs/ml, there is a significant increase in the number of CFUs compared with the control EPCs (8.880 ± 1.22 vs. 6.000 ± 0.50 CFUs/field; *p* < 0.05). To disprove whether this proliferative effect is due to other molecules, the cells were treated with an MP-free supernatant, and we observed no increase in CFU number (5.896 ± 0.5 vs. 6.000 ± 0.5 CFUs/field; *p* = 0.7). In contrast, if EPCs were treated with the MPs from activated cells but at a different concentration (10^5^ MPs/ml), we observed a mechanism contrary to that described with 10^3^ MPs/ml; there was a significant decrease in the number of CFUs compared to the control (2.194 ± 0.36 vs. 6.000 ± 0.50 CFUs/field; *p* < 0.05). Finally, we observed that in EPCs cultures in the presence of TNFalpha (10 ng/ml), CFU formation by the EPCs could not be detected (Figure 3B).

### EPCs treated with MPs from activated endothelial cells are involved in wound healing progression in an endothelial cell monolayer

**Figure 4:**
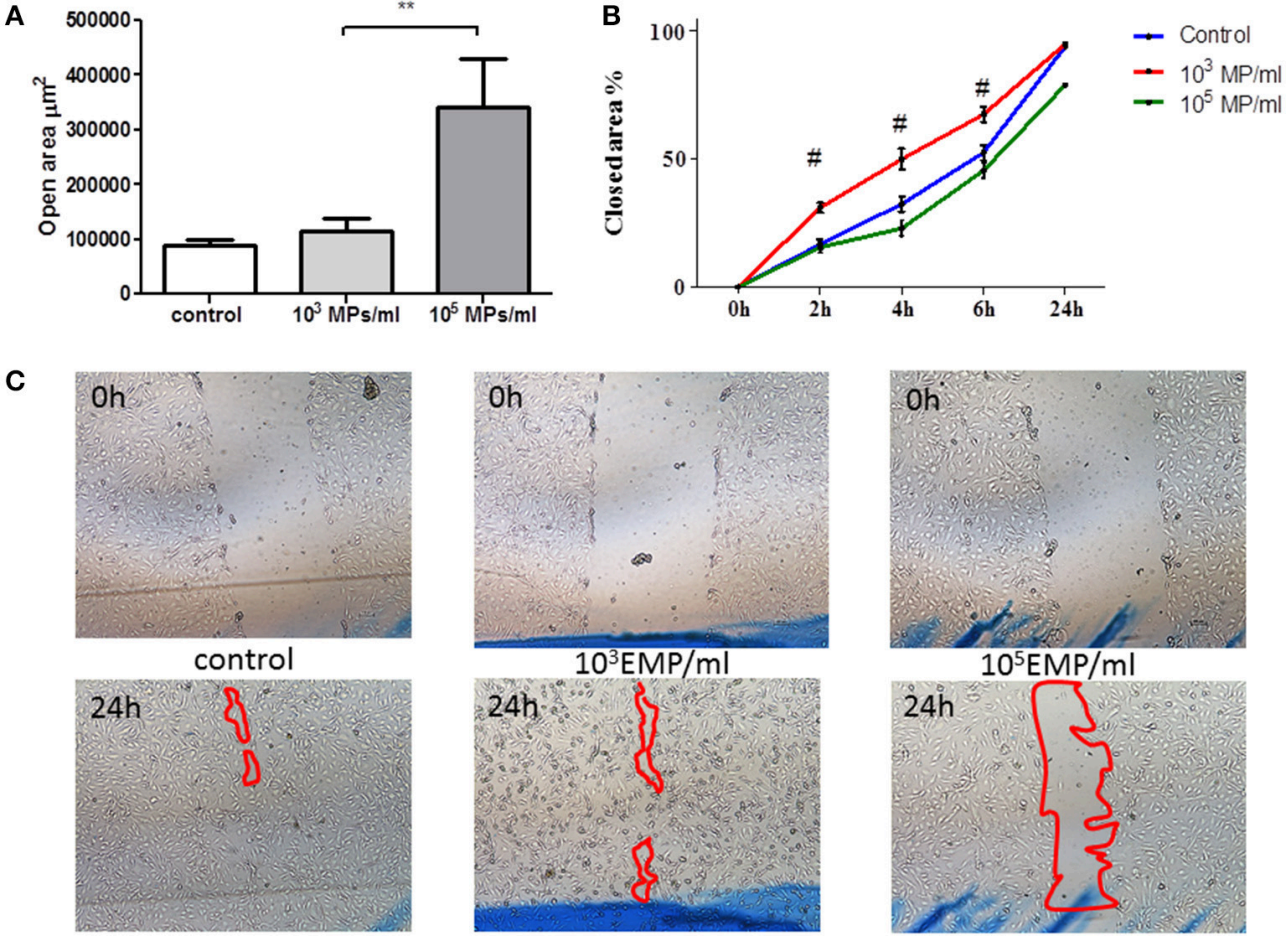
**Wound healing assay in mature HUVECs in the presence of treated EPCs**. **(A)** Area free of cells after 24 h of physical damage to the culture. **(B)** % area closed with respect to the time in which the wound was healing, after contact with treated EPCs on the HUVEC monolayer. **(C)** Representative images at initial time (upper row) and at the final time point of 24 h (lower row) of wound healing by HUVECs in contact with EPCs treated with concentrations of EMPs from the TNFalpha treatments (40 ×). ^**^p < 0.05 vs. Control and 10^3^ MP/ml; ^#^*p* < 0.001 vs. Control and 10^5^ MP/ml.

EPCs treated with TNF MPs (10^3^ MPs/ml) led mature endothelial cells to close the wound at the same rate as the control cells. However, those EPCs treated with the highest dose of MPs (10^5^ MPs/ml) delayed wound healing, leaving behind a cell free zone in the inflicted wound corresponding to 21% of the total initial wound area (Figure 4). The open area remaining at 24 h in mature endothelial cells in contact with EPCs treated with high MP doses (10^5^ MPs/ml) is significantly larger than that left behind from EPCs treated with low MP doses (10^3^ MPs/ml) (341.998 ± 88.779 μm^2^ vs. 115.184 ± 22.766 μm^2^, respectively; *p* = 0.001) (Figure 4).

### EPCs treated with MPs from activated endothelial cells have different adhesion capabilities on an endothelial cell monolayer

**Figure 5:**
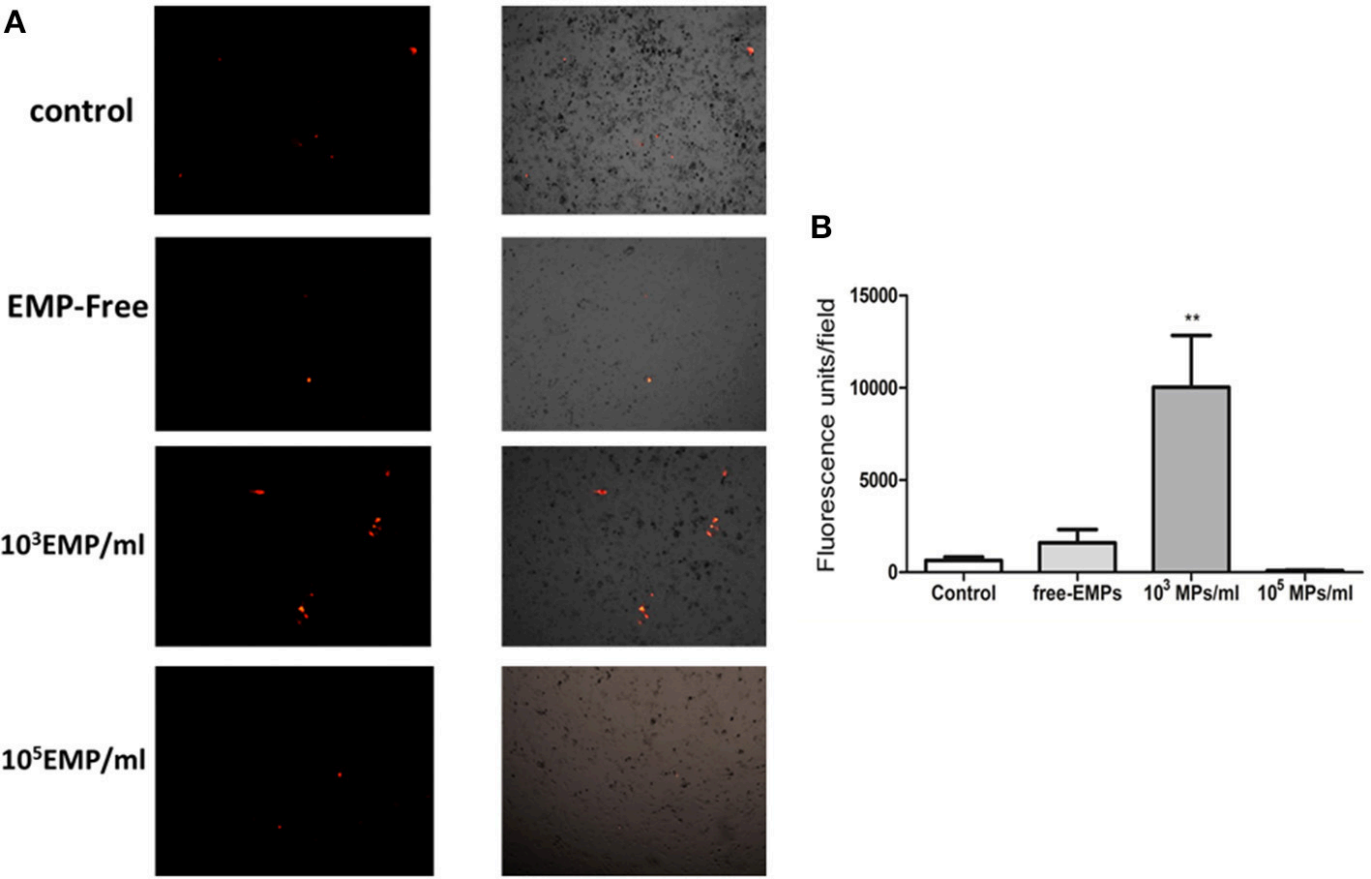
**(A)** Representative inverted microscopy images of the HUVEC monolayer and the adhesion of EPCs treated with different doses of MPs from the TNFalpha cultures, merging the images from the bright field and fluorescence filter for CellTracker™ CM-DiI (40 ×). **(B)** Representative histogram of fluorescent units obtained per field at different endothelial cells plus treated EPCs co-culture conditions. ^**^p < 0.001 vs. Control, free-EMPs and 10^5^ MPs/ml.

We observed that the adhesion capacity of EPCs treated with **a low** MP doses (10^3^ MPs/ml) over 24 h is significantly higher than that of EPCs treated with **a high** doses of MPs (10^5^ MPs/ml) (10.028 ± 2.80 vs. 95.400 ± 26.60 FU/field; *p* < 0.001) (Figure 5). Moreover, to corroborate the absence of any factor other than MPs, we cultivated the EPCs with MP-free supernatants and observed that the adhesion capacity of these cells was slightly higher than that of the control EPCs (1599 ± 721. vs. 643.354 ± 189.1 FU/field; *p* = 0.4). The EPCs treated with higher doses had lower adhesion capacity under all conditions, even presenting a lower adhesion tendency when compared with the control EPCs (95.40 ± 26.6 FU/field vs. all conditions; *p* < 0.001) (Figure 5).

### Pro/anti-angiogenic effects of MP-treated EPCs on mature endothelial cells

**Figure 6:**
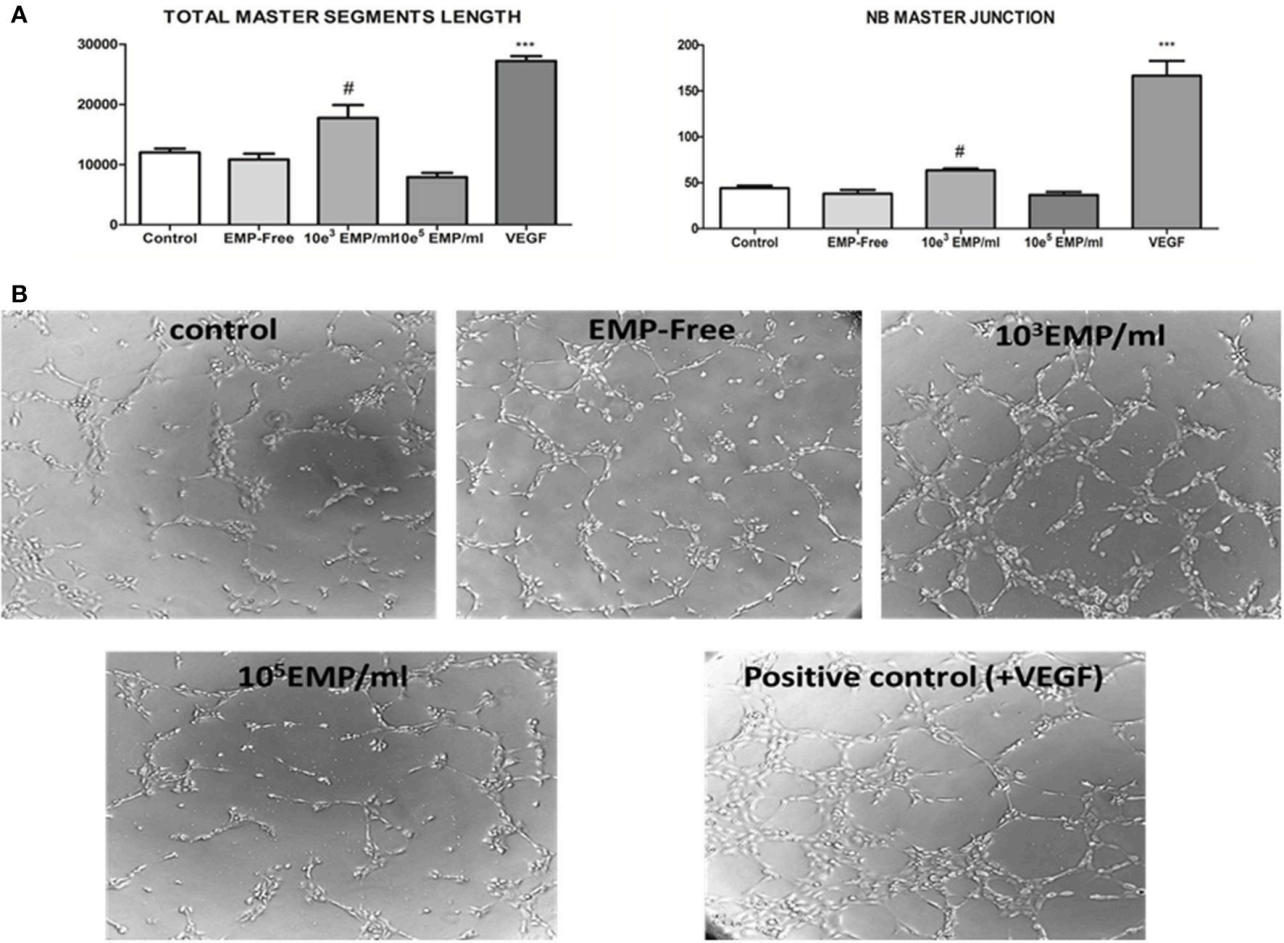
**(A)** (Left) Histogram of the obtained values for the quantification total master segments length parameter for angiogenesis; (right) for another parameter defined as the NB master junction. ^#^*p* < 0.05 vs. all conditions; ^***^p < 0.001 vs. all conditions. **(B)** Representative inverted optical microscopy images of the formation of vessels in the *in vitro* angiogenesis assay on Matrigel at the final time point (3 h) (40 ×).

We evaluated the capacity of EPCs to induce angiogenesis in mature endothelial cells, and observed their capacity to stimulate angiogenesis when the EPCs were treated with a low MP dose (10^3^ MPs/ml) compared with the control (17,749 ± 1505 μm vs. 12024 ± 456 μm *p* < 0.05). However, we observed that EPCs treated with a high dose of MPs (10^5^MPs/ml) lose some of this capacity, such that mature endothelial cells form a significantly lower number of vascular vessels in the 3D matrix for angiogenesis (7913 ± 499 μm vs. 12024 ± 456 μm; *p* < 0.05) (Figure 6). Moreover, we also confirmed that this mechanism is dependent on the treatment of the EPCs with MPs because those EPCs treated with MP-free supernatants behave similar to the control EPCs (10834 ± 689 μm vs. 12024 ± 456 μm; *p* = 0.35). Last, it must be highlighted that the induction of vascular vessel formation in mature endothelial cells via EPCs treated with 10^3^MPs/ml produces 55% of the total capacity of these cells regarding the formation of vessels in the presence of VEGF (Figure 6).

## Discussion

Vascular endothelial cells are constantly exposed to factors such as cytokines (Mackay et al., 1993) or different toxins (Dou et al., 2002) circulating in the bloodstream that may provoke endothelial cell damage or death (Liu et al., 2015); therefore, there is a physiological turnover mechanism for endothelial cells mainly associated with the activation and differentiation of progenitor endothelial cells (Zhang et al., 2014b). In this study we show how EPCs proliferate, differentiate and acquire the capacity to bind to endothelial cells in response to stimulation by MPs produced by TNFalpha-activated endothelial cells.

Damage produced by TNFalpha to mature endothelial cells is reflected in the higher expression of endothelial cell adhesion markers (ICAM, PECAM) (Swerlick et al., 1992). At 72 h, a decrease in the MFC can be observed for these membrane proteins, which could be explained by the greater number of apoptotic cells and lower number of proliferating cells at this time point, which represent additional impacts of the long-term damage induced by TNFalpha (Deshpande et al., 2000). A lot of works demonstrate that TNFalpha is a good stimulus to activate endothelial cells (Zhou-Stache et al., 2002). There are a great variety of signaling pathway of TNFalpha treated cells (Yan et al., 2011). Moreover, some works propose that the endothelial activation by TNFalpha increase the release of microRNAs loading microparticles (Zhang et al., 2014a). In our model, we observed that there is a time that was considered as activation time (24 h). In this point, the microparticles released were used to modify EPCs culture in a dose-dependent manner.

Regarding the expression of EMPs in culture, we observed that the event/μl density assessed by the flow cytometer is higher than under baseline conditions, which leads us to suggest that in response to endothelial activation by TNFalpha, the cells are capable of releasing a higher number of MPs into the medium as a result of the damage induced by the stimulus. We hypothesized that different quantity of EMPs could act as different signaling process between cells; in our work we focused on the interaction of MPs with EPCs. Until now, the works only characterize the microparticles and/or utilize cells lines well-knowns. We used EPCs that were isolated from peripheral blood and we tried to assess the functionally changes of these *in vitro*.

Our hypothesis is on the way that EMPs could carry information to recruit EPCs from bone narrow to mobilize them and repair the endothelial damage made by multiple factors in a pathologies context.

EPC progression *in vitro* depends on the dose of MPs from TNFalpha-stimulated endothelial cells. High doses (10^5^ MPs/ml) attenuate the formation of CFUs in a fibronectin matrix during 25–30 days of culture. In contrast, low doses (10^3^ MPs/ml) enhance CFU formation compared with both the control and MP-free conditions.

Regarding the healing of an exogenously inflicted wound in a mature endothelial cell culture, the treatment of EPCs with a high dose of MPs (10^5^ MPs/ml) causes a loss of the cooperative function of the EPCs in closing the wound.

Consistent with the wound closing experiment, the EPCs treated with a high MP dose (10^5^ MPs/ml) lose some of their ability to adhere to the mature endothelial cell monolayer.

We suggest that those EPCs treated with high doses of MPs (10^5^ MPs/ml) lose a portion of their angiogenic potential on mature endothelial cells and that those treated with low doses (10^3^ MPs/ml) enhance this effect.

Of special interest is the observation that to induce a repair response via the EPCs, the MPs must be at a low proportion (approximately 1 MP per 100 cells), which could explain the lack of repair activity by EPCs described in pathologies that present significant endothelial damage or low production of EPCs.

In response to the inflammatory onslaught induced by the cytokine TNFalpha, endothelial cells produce MPs (Alexy et al., 2014). In agreement with previously published studies (Mezentsev et al., 2005), when adding these MPs to EPCs, we observe increased cell proliferation as well as an increase in the capacity of EPCs to adhere to the damaged endothelium. The dose-response study indicated that MPs induce proliferation and adhesion in EPCs at relatively low doses (10^3^ MPs/ml), a dose that is commonly observed in the plasma of healthy individuals (Clarke et al., 2010). A reduced proliferation and adhesion of EPCs was observed when using 10^5^ MPs/ml. We consider these results interesting because, until now, the use of MPs as markers of endothelial damage has been limited to merely demonstrating an increase in the number of these MPs in the plasma associated with different pathologies (Chironi et al., 2009). However, our study suggests that at least to activate the functionality of EPCs, the number of MPs is as important as the availability of target cells. That is, a high number of EMPs would be useful in stimulating EPCs activity when these EMPs are in contact with EPCs that can be mobilized, such as in a healthy individual. However, in elderly people or during a pathological condition in which a decreased number of EPC cells has been described (Chang et al., 2007; Williamson et al., 2013; Castejon et al., 2014), the same amount of EMPs would not only prevent the activity of these cells but would also inhibit it.

The specificity of a mechanism that is dependent on the stimulation of EPC capacity by MPs was revealed when we demonstrated that MP-free supernatant was incapable of inducing the proliferation and/or adhesion of EPCs.

In summary, our study identifies the MPs produced by activated endothelial cells as intercellular signaling factors that induce EPCs to proliferate, differentiate, adhere and regenerate the damaged endothelium. In addition, our results suggest that this activity is associated with the number of MP that has been added to the target cells. When the ratio of MPs is approximately 2:100 MPs/target cells, these MPs are able to stimulate the different competence of this cells *in vitro*. However, when the MPs are approximately in ratio 1:1, occurs the opposite fact, and the EPCs lose their abilities. In pathological conditions there is an increase of total MPs, a decrease of circulating EPCs, or both. Therefore, the ratio would be proportionally modified.

These results reinforce previous studies suggesting the use of MPs as components of vascular regeneration therapy. The results also expand the potential of MPs therapy by indicating that the number of circulating MPs is key role in the repair activity of EPCs; thus, new potential therapeutic strategies could be directed at decreasing the elevated number of circulating MPs that has been described in patients with vascular disease.

Our study could provide a foundation for designing possible therapeutic strategies utilizing MPs as activating factors in vascular repair and regeneration, taking into accounts the population of circulating EPCs in pathological conditions and the specific expression of MPs in these same pathologies.

## Author contributions

Each author has contributed significantly to the submitted work: Conceived and designed the experiments: RR, JC; performed the experiments: CL, AC; analyzed the data: CL, AC, MA wrote the paper: CL, AC, RR.

### Conflict of interest statement

The authors declare that the research was conducted in the absence of any commercial or financial relationships that could be construed as a potential conflict of interest.
